# The enzymatic degradation of ATP to adenosine by microglial CD39 regulates neurovascular coupling and metabolic supply to the brain

**DOI:** 10.1007/s11302-025-10102-w

**Published:** 2025-06-20

**Authors:** Jing Guo, Yong Tang, Peter Illes

**Affiliations:** 1 International Research Center on Purinergic Signaling, School of Acupuncture and Tuina, Chengdu University of Traditional Chinese Medicine, Chengdu, China; 2 Acupuncture and Chronobiology Key Laboratory of Sichuan Province, Chengdu University of Traditional Chinese Medicine, Chengdu, China; 3 School of Health and Rehabilitation, Chengdu University of Traditional Chinese Medicine, Chengdu, China; 4 Rudolf Boehm Institute for Pharmacology and Toxicology, University of Leipzig, Leipzig, Germany

**Keywords:** Adenosine 5′-triphosphate, Cerebral blood flow, CD39

## Article summary

In a recent paper published in *Nature Communications*, Fu et al. provided evidence that microglial CD39 is essential for the generation of adenosine from the (co)transmitter adenosine 5′-triphosphate (ATP), which then activates vasodilatory A_2A_ receptors (A2ARs) at the cerebral vasculature to increase cerebral blood flow (CBF) [1]. CBF is, among other processes, regulated by adenosine via A2ARs to satisfy the brain’s energy demand for glucose/nutrient and oxygen supply during phases of altered neuronal activity. Diverse pharmacological and genetic approaches aimed at ablating microglial populations, identified the microglial enzyme, CD39, as the key target of this purinergic regulatory mechanism. The following experimental approaches were used: (a) PLX3397 reversibly depleted microglia; (b) the crossing of CXCR1-CreER and CAG-26R-TDA mice resulted in ablation of microglia in their offspring, due to damage via the expression of diphtheria A-toxin; and (c) fms-intronic regulatory element (FIRE)–deleted mice showed selective microglial depletion, but no change in the number of perivascular macrophages. All of these manipulations caused the blunting of whisker stimulation (stimulus for the release of ATP/adenosine)–induced increases of CBF in the barrel cortex. The genetic deletion of CD39 and its pharmacological blockade by ARL67156 both blocked the effect of whisker stimulation. In conclusion, CD39 appears to be the key enzyme in degrading neuronal ATP to adenosine and thereby exerts a feedback control imposed on the neurovascular tone regulating the delivery of nutrients and oxygen to the brain.

## Commentary

Perivascular adenosine A2ARs are known to regulate neurovascular coupling, connecting neuronal activity to CBF that supplies glucose and oxygen to satisfy the brain’s energy demands. ATP is a (co)transmitter released from the terminals of central nervous system neurons. This ATP is sequentially degraded by ectonucleotide tri(di)phosphohydrolase (ENTPD; CD39) to AMP, which is then transformed by ecto-5′-nucleotidase (ENT5; CD73) to adenosine [2]. Microglial cells are resident immunocytes maintaining the brain’s homeostasis by phagocytosing pathogenic bacteria and endogenous waste, as well as repairing the blood–brain barrier in the case of injury [3]. Microglia and vascular endothelial cells are also the exclusive source of CD39 in the brain. Microglia detect ATP in the central nervous interstitium via P2Y_12_ receptors (P2Y12Rs); ATP, flowing out from the cytoplasm of damaged cells, causes the extension of microglial processes after its recognition by this receptor and thereby enables microglial phagocytosis.

Adenosine generated from ATP has previously been shown to act via A2ARs to increase CBF and so regulate metabolic processes [4]. This is a further function of adenosine generated by microglial CD39 that depends on the release of neuronal ATP, as it is also known to exert feedback inhibition of network activity through neuronal A1Rs. The aim of the present study was to clarify whether CD39 or CD73 of microglia is involved in the regulation by ATP-derived adenosine, promoting CBF. A range of convincing experimental approaches confirmed the notion that CD39 is the primary player involved in this event.

The present paper utilized numerous approaches to procure microglial ablation or delete microglial CD39. These were (a) the reversible inhibition of the colony-stimulating factor 1 (CSF1R) receptor by PLX3397 (pexidartinib) and the consequent reversible depletion of microglia; (b) the genetic ablation of microglia by crossing CXCR1-CreER and CAG-R26R-DTA mice and administering tamoxifen to express the injurious diphtheria A-toxin in attachment with the chemokine receptor 1 (CXCR1) in their offspring; and (c) the use of CSF1R super-enhancer FIRE-deleted mice. This latter type of transgenic animal had the advantage of selective depletion of microglia, without altering the number of perivascular macrophages, which are also brain-resident mononuclear cells that, similar to microglia, contribute to phagocytosis and brain homeostasis [5]. Finally, (d) CX3CR1 and *Entpd1* (CD39)-floxed mice, as well as *Nt5e* (CD73)-floxed mice, were crossed to genetically inactivate CD39 and CD73, respectively, in microglia.

In all following experiments, the participation of microglia or their ATP-degrading enzymes/ATP-sensitive receptors was investigated in the mediation of neurovascular responses (rise in CBF) to endogenously released (whisker-stimulation) or exogenously applied (intra-cisternal injection) ATP/adenosine. The measurement by fast-scan cyclic voltammetry of adenosine concentrations in the brain confirmed the massive upsurge of this vasoactive purine molecule in the barrel cortex in response to whisker stimulation.

Laser speckle contrast imaging was used to compare the baseline and mouse whisker stimulation–induced increase in CBF. In this series of experiments, microglia were ablated by treatment with the CSFR1 inhibitor, PLX3397. This compound has a reversible effect; therefore, CBF could be determined before, during and after treatment with this drug. It was found that PLX3397 elevated the CBF baseline and blunted the contralateral response to whisker stimulation, without altering the peak level of CBF. A differential effect of microglial ablation on basal and stimulation-induced changes in CBF was unexpected, because, as reported later, both the intra-cisternal injection of adenosine and whisker stimulation resulted in a uniform decline of the vascular tone, measured as CBF increase. All effects were reversible and consistent with the CBF responses to hypercapnia instead of whisker stimulation.

Chemogenetic manipulation of G_q_- or G_i_-coupled designer receptors exclusively activated by designer drugs (DREADD) of microglia showed that acute G_q_ activation induced prolonged elevation of CBF, whereas G_i_ activation had no obvious effect. G_q_ activation also blunted CBF responses to whisker stimulation. These results are in favor of a restricted neurovascular coupling reserve by microglial ablation. The involvement of a G_q_-coupled receptor in this process is consistent with the transduction pathway of A2ARs, suggested to be the target of adenosine in the cerebral vasculature.

Bulk RNA sequencing allowed the comparison of the cerebral cortical transcriptome of microglia-containing and microglia-ablated mice under control conditions and PLX3397-treatment conditions. In the latter group of animals, microglial signature markers (e.g., *Tmem119*,* P2ry12*,* Cx3crl*, *Trem2*) were significantly decreased, as were *Entpd1* for CD39 and *Ptgs1* for COX1. By contrast, the depletion of microglia had minimal effects on many other vasoactive genes.

An important and most conclusive series of experiments demonstrated that the injection of ATP into the cisterna magna induced a slower, but higher rise in CBF than did adenosine. It was also reported that while microglial ablation by PLX3397 markedly blunted the effect of ATP, it did not alter that of adenosine. Hence, adenosine itself was shown to be responsible for the increase in CBF, in contrast to ATP, which had to be degraded by microglial CD39 before becoming effective. Another set of data also suggested that ATP itself is not the mediator of the vascular response. When an enzymatically stable structural analogue of ATP, ATP-γ-S, was injected intra-cisternally, a biphasic effect on CBF developed (reduction followed by increase), invariably leading to animal death.

The next question to be addressed was whether parenchymal microglia or perivascular macrophages mediate the described neurovascular coupling process. FIRE-deleted mice were used to do so. After confirming an almost complete absence of Iba1^+^-immunopositive microglia in the brain of this type of animal, a rise in the basal CBF and a reduction in the CBF response to whisker stimulation were observed in the FIRE mice when compared with their wild-type (WT) controls. Further, FIRE mice showed diminished CBF rises in response to intra-cisternal ATP, in contrast to their WT counterparts. Another defining characteristic of parenchymal microglia (but not perivascular macrophages) is the presence of the chemotactic P2Y12R. Consistent with this notion, a diminished CBF reactivity to whisker stimulation was found in the P2Y12R knockout mice compared with the respective WT control mice.

The inhibition of CD39 by ARL67156 also led to an increase of the basal CBF and a blockade of the effects of whisker stimulation, similar to the effect of microglia depletion by various procedures. Genetic deletion of microglial CD39 raised the basal CBF and blunted responses to whisker stimulation and intra-cisternal ATP, but not adenosine, whereas genetic deletion of CD73 resulted in similar responses to whisker stimulation and ATP injection into the cisterna magna.

In conclusion, a combination of pharmacological and genetic approaches to ablate microglia unequivocally suggests that these immunocytes are involved in the mediation of endogenously released or intra-cisternally applied ATP, but not adenosine, on increasing CBF, an indication of enhanced neurovascular activation. These findings, in addition, showed that parenchymal microglia rather than perivascular macrophages and CD39, rather than CD73 are the mediators of the neurovascular regulation of metabolic homeostasis.

This extensive and convincing study has only three incompletely resolved parts. (a) The adenosine levels elevated by whisker stimulation were determined, but the concomitant ATP levels were neglected, although these experiments are absolutely essential for the evaluation of the whisker-stimulation data. (b) The authors fail to supply an explanation for the differential change in the basal and whisker stimulation–induced CBF responses by general (PLX3397; diphtheria toxin) and selective (FIRE mice; P2Y12R KO mice) microglial ablation. This could be due to methodological reasons or to the way of presenting these results, but an explanation would be helpful. (c) It is quite clear that microglia might use also other effectors beyond CD39 to exert effects on CBF, such as vasodilatory arachidonic acid metabolites. These effects could be of considerable importance, since the blockade of CD39 by ARL67156 and the genetic deletion of P2Y12R left a rather large fraction of the CBF response to whisker stimulation unaltered.

## Data Availability

No datasets were generated or analysed during the current study.
